# Mercury and arsenic attenuate canonical and non-canonical NLRP3 inflammasome activation

**DOI:** 10.1038/s41598-018-31717-7

**Published:** 2018-09-12

**Authors:** Huijeong Ahn, Jeongeun Kim, Seung Goo Kang, Sung-il Yoon, Hyun-Jeong Ko, Pyeung-Hyeun Kim, Eui-Ju Hong, Beum-Soo An, Eunsong Lee, Geun-Shik Lee

**Affiliations:** 1 College of Veterinary Medicine and Institute of Veterinary Science, Kangwon National University, Chuncheon, Gangwon 24341 Republic of Korea; 2 Department of Molecular Bioscience, School of Biomedical Science, Kangwon National University, Chuncheon, Gangwon 24341 Republic of Korea; 3 Division of Biomedical Convergence, College of Biomedical Science, Kangwon National University, Chuncheon, Gangwon 24341 Republic of Korea; 4 Laboratory of Microbiology and Immunology, College of Pharmacy, Kangwon National University, Chuncheon, Gangwon 24341 Republic of Korea; 5 College of Veterinary Medicine and Institute of Veterinary Science, Chungnam National University, Daejeon, 34134 Republic of Korea; 6 Department of Biomaterial Science, College of Natural Resources and Life Science, Pusan National University, Gyeongsangnam-do, 50612 Republic of Korea

## Abstract

Exposure to heavy metals can cause several diseases associated with the immune system. Although the effects of heavy metals on production of inflammatory cytokines have been previously studied, the role of heavy metals in inflammasome activation remains poorly studied. The inflammasome is an intracellular multi-protein complex that detects intracellular danger signals, resulting in inflammatory responses such as cytokine maturation and pyroptosis. In this study, we elucidated the effects of four heavy metals, including cadmium (Cd), mercury (Hg), arsenic (As), and lead (Pb), on the activation of NLRP3, NLRC4, and AIM2 inflammasomes. In our results, mercury and arsenic inhibited interleukin (IL)-1β and IL-18 secretion resulting from canonical and non-canonical NLRP3 inflammasome activation in macrophages and attenuated elevation of serum IL-1β in response to LPS treatment in mice. In the mechanical studies, mercury interrupted production of mitochondrial reactive oxygen species, release of mitochondrial DNA, and activity of recombinant caspase-1, whereas arsenic down-regulated expression of promyelocytic leukemia protein. Both mercury and arsenic inhibited Asc pyroptosome formation and gasdermin D cleavage. Thus, we suggest that exposure to mercury and/or arsenic could disrupt inflammasome-mediated inflammatory responses, which might cause unexpected side effects.

## Introduction

Heavy metals such as cadmium (Cd), mercury (Hg), arsenic (As), and lead (Pb) have high atomic weights and densities, and heavy metal poisoning is an ongoing global health problem^1^. Arsenic is regarded as a heavy metal, although it is a metalloid not a metal^1^. Based on emerging data, heavy metal exposure has a negative influence on health and induces neurodegenerative diseases^2^, metabolic syndrome^3^, cancer^1^, and immune disorders^4–7^. In addition, heavy metal poisoning disrupts our immune system and causes increased susceptibility to infection^8^.

Cadmium is present in plastics, batteries, paints, and cigarette smoke. The most well-known outcome of cadmium poisoning is known as “itai-itai disease” presenting severe pain. Cadmium also induces disruption of DNA repair and cellular redox systems^9^. In the immune system, cadmium was reported to induce atrophy of the thymus in a rodent model, alteration of T and B lymphocytes counts, disruption of the humoral and cellular immune responses in animal models, and alteration of cytokine expression in lung cells^9,10^. Mercury contamination mainly occurs via inhalation of mercuric vapor and consumption of foods such as fish and livestock, which accumulate methylmercury. Mercury enters the body and travels to the brain, spinal cord, and peripheral neurons acting as a primary repository of mercury, although symptoms resulting from mercury contamination are induced in other organs^11^. Mercury also induces DNA damage, oxidative stress, and mitochondrial dysfunction at the cellular level and contributes to cardiomyopathy, chest pain, angina, anemia, digestive disturbances, and kidney damage^11^. Mercury is also known to disrupt the immune system via deletion of polymorphonuclear leukocytes, which take part in the clearance of dangerous substances^12^. Mercury has been shown to reduce proliferation of monocytes via inhibition of interleukin (IL)-1 production^13^. Although mercury has been associated with numerous autoimmune diseases, the effects of mercury on the immune response remain controversial^11^. Lead, mainly contained in leaded gasoline, pottery, boat building materials, lead-based paints, pigments, and battery, enters the body via inhalation, ingestion, or skin absorption. Lead poisoning damages the brain, resulting in interference of synapse formation in the cerebral cortex during childhood. Lead also induces nephropathy, abortion, colic-like abdominal pains, bone weakness, and anemia^14^. In the immune system, lead has been shown to significantly alter on the proliferation and activation of T and B lymphocytes and macrophages, but its effects are dependent on dosage and treatment period during proliferation^15^. Arsenic, which is present in contaminated water, cigarette smoke, foods, industrial products, and polluted air, is well-known to induce skin, lung, and bladder cancers and is involved in formation of skin lesions, diabetes, and cardiovascular diseases^6,16^. Arsenic enters the body and is modified into di-methylated arsenicals, which enhance its toxicity^6^. Arsenic not only induces oxidative stress, inhibition of DNA repair, apoptosis, and gene expression alterations but also has deleterious effects on the immune system^6^. Arsenic exposure has been shown to down-regulate the expression of genes related to T-cell receptor signaling, cell cycle, apoptosis, defense, and the immune response, including several cytokines, IL-1β, −2, −4, −5, −10, interferon (INF)-γ, and tumor necrosis factor (TNF)α^6,17^. However, opposite results has been reported as well^18^.

Inflammation is an essential process for the removal of damaged cells and tissue and for initiation of tissue repair. IL-1β and IL-18, the most important cytokines for inducing inflammation, are maturated by inflammasomes, which are intracellular multi-protein complexes^19,20^. Inflammasomes activate caspase-1, which then catalyzes proteolytic cleavage of IL-1β and IL-18 precursors to induce inflammatory cell death pyroptosis^21^. The maturated cytokines, which lack an N-terminal secretion signal^22^, are secreted via gasdermin D (Gsdmd) pore-forming and/or pyroptosis^23^. Inflammasomes consist of caspase-1 and Asc (apoptosis-associated speck-like protein containing a caspase recruitment domain [CARD]) and trigger sensing proteins such as Nucleotide-binding oligomerization domain (NOD), Leucine-rich Repeat and Pyrin domain containing protein 3 (NLRP3), NOD-like receptor (NLR) family CARD-containing protein 4 (NLRC4), and absent in melanoma 2 (AIM2). NLRP3 recognizes endogenous and exogenous danger signals such as adenosine triphosphate (ATP), monosodium urate crystals (MSU), and nigericin (bacterial toxin), and NLRP3 inflammasome is triggered by non-canonical inflammasome, which is activated by intracellular lipopolysaccharide (LPS)^24–27^. NLRC4 inflammasome is stimulated by intracellular flagellin and conserved type 3 secretion system rod component (T3SS) derived from bacteria^28^. AIM2 inflammasome senses double-stranded DNA (dsDNA)^29^. These inflammasomes, which exist in myeloid and non-myeloid cells such as monocytes, macrophages, neutrophils, dendritic cells, fibroblasts, heart, kidney, and intestinal epithelial cells, are a crucial immune response against cytosolic danger signals^26^.

Although several reports have reported the immuno-toxicological effects of heavy metals on cytokine production, precise investigation of the effects of heavy metals on inflammasome activation has not been carried out. In this study, we elucidated the effects of heavy metals such as cadmium, mercury, arsenic, and lead on the activation and priming steps of inflammasomes in macrophages. We also tested the role of heavy metals in triggering activation of NLRP3, NLRC4, and AIM2 inflammasomes. Finally, we assessed the intracellular inhibitory mechanism responsible for the effects of heavy metals on inflammasome activation.

## Results

### Mercury and arsenic inhibit activation of NLRP3 inflammasome

**Figure 1:**
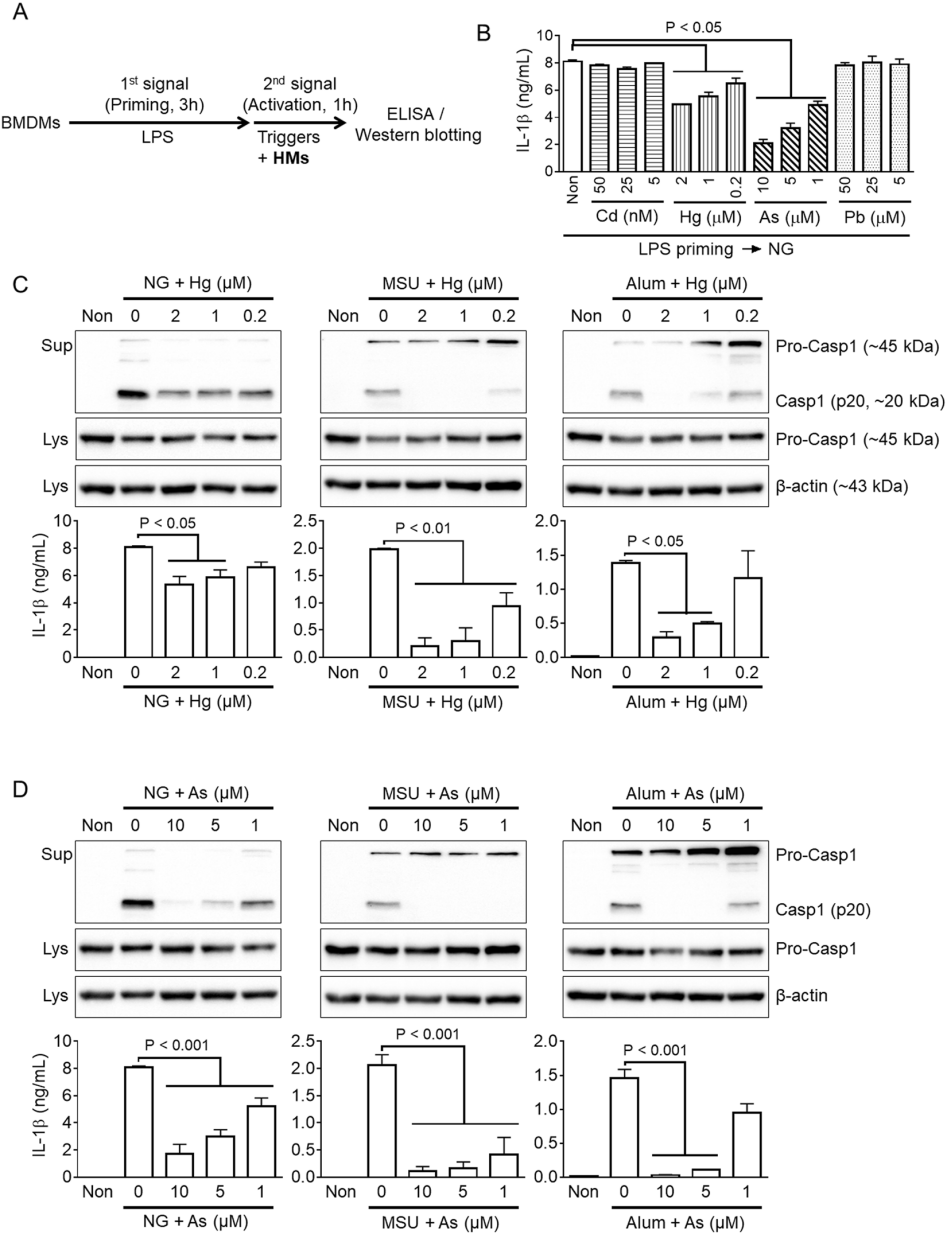
Effects of heavy metals on NLRP3 inflammasome activation. (**A**) Schematic diagram for inflammasome activation. BMDMs (1 × 10^6^ cells per well) were treated with LPS (1 μg/ml) as a 1^st^ signal for priming, after which cells were replaced with media containing an inflammasome trigger (2^nd^ signal) with/without heavy metals (HMs) as indicated. Inflammasome activation was assessed by ELISA and/or immunoblotting. (**B**) LPS-primed BMDMs were treated with nigericin (NG) in the presence of media only (Non), cadmium (Cd), mercury (Hg), arsenic (As), or lead (Pb). Release of IL-1β was analyzed by ELISA. (**C**) LPS-primed BMDMs were subjected to activation of NLRP3 inflammasome by NG, monosodium urate crystal (MSU), or aluminum (Alum) treatment in the presence of an increasing dosage of mercury (Hg). The cleaved form of caspase-1 (Casp1, p20) was detected by immunoblotting, and secretion of IL-1β was observed with ELISA. (**D**) LPS-primed BMDMs were treated with NG, MSU, or Alum in the presence of arsenic (As). Release of Casp1 and IL-1β was analyzed with immunoblotting and ELISA. All immunoblot data shown are representative of at least three independent experiments. Bar graph presents the mean ± SD with at least two independent experiments.

To elucidate the effects of heavy metals, cadmium (Cd), mercury (Hg), arsenic (As), and lead (Pb), on inflammasome activation, BMDMs were primed with LPS to induce expression of inflammasome components such as pro-IL-1β and NLRP3, followed by treatment with nigericin (NG) to trigger activation of NLRP3 inflammasome, as shown in Fig. 1A. IL-1β secretion, a readout of inflammasome activation, was measured by ELISA (Fig. 1B). Mercury and arsenic significantly attenuated release of IL-1β resulting from NLRP3 inflammasome activation in a dose-dependent manner, whereas cadmium and lead did not. We further confirmed the anti-NLRP3 inflammasome activities of mercury and arsenic using other NLRP3 triggers, MSU and aluminium crystal (Alum). LPS-primed BMDMs were treated with increasing dosages of mercury or arsenic in the presence of NG, MSU, or Alum. As shown in Fig. 1C, mercury attenuated caspase-1 cleavage, another readout of inflammasome activation, as well as inhibited IL-1β secretion resulting from NG, MSU, or Alum-mediated NLRP3 inflammasome activation. Arsenic presented similar anti-NLRP3 properties as mercury. Arsenic blocked secretion of caspase-1 and IL-1β in response to NG, MSU, or Alum (Fig. 1D). We further confirmed the effects of heavy metals on inflammasome-mediated cell death since inflammasome activation induces caspase-1 mediated cell death, known as pyroptosis^21^. Consistent with secretion of IL-1β, mercury and arsenic inhibited pyroptosis in BMDMs (Supplemental Fig. 1A,B). The current concentrations of mercury and arsenic did not present any cytotoxicity in BMDMs (Supplemental Fig. 1C,D). These results suggest that mercury and arsenic interrupted NLRP3 inflammasome activation.

### Mercury and arsenic inhibit non-canonical NLRP3 inflammasome

**Figure 2:**
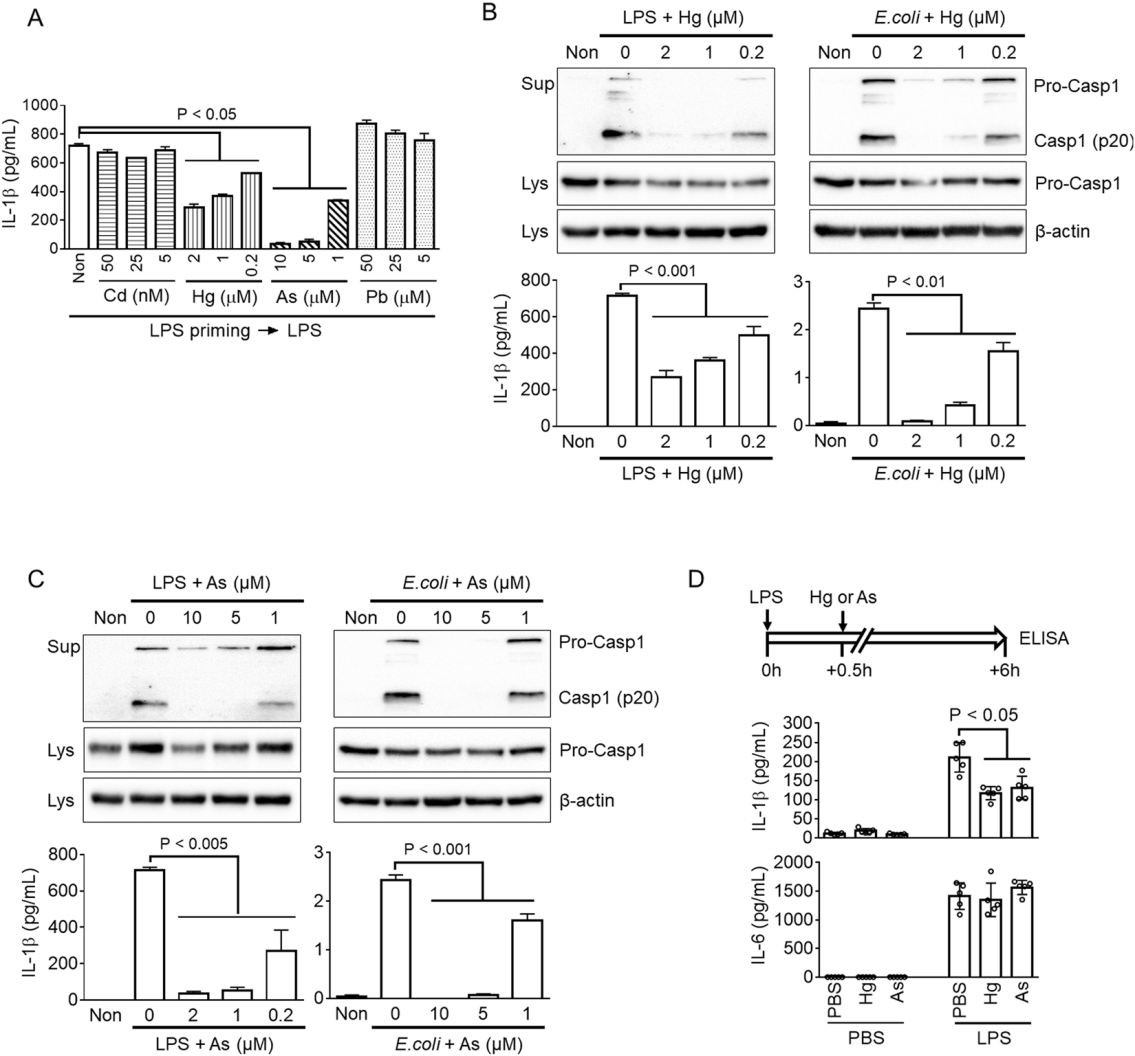
Effects of heavy metals on non-canonical NLRP3 inflammasome activation. (**A**) LPS-primed BMDMs were transfected with LPS to activate non-canonical inflammasome in the presence of media only (Non), cadmium (Cd), mercury (Hg), arsenic (As), or lead (Pb). Release of IL-1β was analyzed by ELISA. (**B**) LPS-primed BMDMs were subjected to activation of NLRP3 inflammasome activation via the non-canonical pathway using LPS transfection (left panel) or *E. coli* infection (right panel) in the presence of an increasing dosage of mercury (Hg). The cleaved form of caspase-1 (Casp1, p20) was detected by immunoblotting, and secretion of IL-1β was observed with ELISA. (**C**) LPS-primed BMDMs were treated with LPS or *E. coli* with/without arsenic (As). Release of Casp1 and IL-1β was detected by immunoblotting or ELISA. Mice (n = 5 per group, total n = 30) were intraperitoneally administrated with PBS, mercury, or arsenic 30 min after ip injection of PBS or LPS. After an additional 5.5 h, serum IL-1β and IL-6 were analyzed by ELISA. All immunoblot data shown are representative of at least three independent experiments. Bar graph presents the mean ± SD with at least two independent experiments.

We further tested the effects of heavy metals on NLRC4 and AIM2 inflammasomes and the non-canonical pathway of NLRP3 inflammasome. LPS-primed BMDMs were transfected with flagellin or dsDNA to activate NLRC4 and AIM2 inflammasomes, and IL-1β secretion was measured by ELISA (Supplemental Fig. 2A,B). For the results, none of the heavy metals altered IL-1β secretion resulting from NLRC4 and AIM2 inflammasome activation. Next, we transfected LPS into the cytoplasm of BMDMs, which interact with caspase-11 to cause activation of NLRP3 inflammasome^30,31^. IL-1β release in response to LPS transfection was observed in the presence of heavy metals (Fig. 2A). Mercury and arsenic significantly attenuated NLRP3 inflammasome-mediated IL-1β secretion resulting from non-canonical inflammasome activation. To confirm the effects of mercury and arsenic on non-canonical inflammasome activation, LPS-primed BMDMs were infected with *E.coli*, another trigger for non-canonical inflammasome^30^. For the results, mercury attenuated maturation of caspase-1 and IL-1β, as shown in Fig. 2B. In addition, arsenic blocked LPS- and *E.coli*-mediated non-canonical inflammasome activation (Fig. 2C). Thus, mercury and arsenic interrupted both the canonical and non-canonical pathways of NLRP3 inflammasome activation.

We further confirmed whether or not heavy metals attenuate inflammasome activation in human cells. We adopted a human monocyte-like cell line, THP-1, which were differentiated into macrophage-like cells by phorbol 12-myristate 13-acetate (PMA) treatment. PMA-treated THP-1 cells were primed by LPS and then subjected to inflammasome activation similar to murine BMDMs (Fig. 1A). For the results, mercury and arsenic inhibited IL-1β secretion in response to NG (Supplemental Fig. 3A) and MSU (Supplemental Fig. 3B), whereas cadmium and lead did not. Activation of non-canonical inflammasome, which was trigged by LPS transfection, was also blocked by mercury and arsenic treatment in human cells (Supplemental Fig. 3C). However, NLRC4 and AIM2 inflammasome activations were not altered by heavy metal co-treatment in contrast to mouse BMDMs (Supplemental Fig. 3D,E). Taken together, mercury and arsenic inhibited NLRP3 and non-canonical inflammasomes in both human and mouse macrophages.

Next, the inhibitory effects of mercury and arsenic on NLRP3 inflammasome activation were assessed in an animal study. Mice were intraperitoneally injected with PBS, mercury, or arsenic 30 min after LPS treatment, and serum IL-1β and IL-6 levels were analyzed as shown in the upper panel of Fig. 2D. IL-β secretion resulting from LPS-mediated NLRP3 inflammasome activation was significantly attenuated by mercury and arsenic treatments while LPS-mediated IL-6 release was not altered (Fig. 2D). Taken together, mercury and arsenic not only inhibited NLRP3 inflammasome in BMDMs but also attenuated IL-1β secretion in an animal model.

### Arsenic inhibits the priming step of inflammasome activation

**Figure 3:**
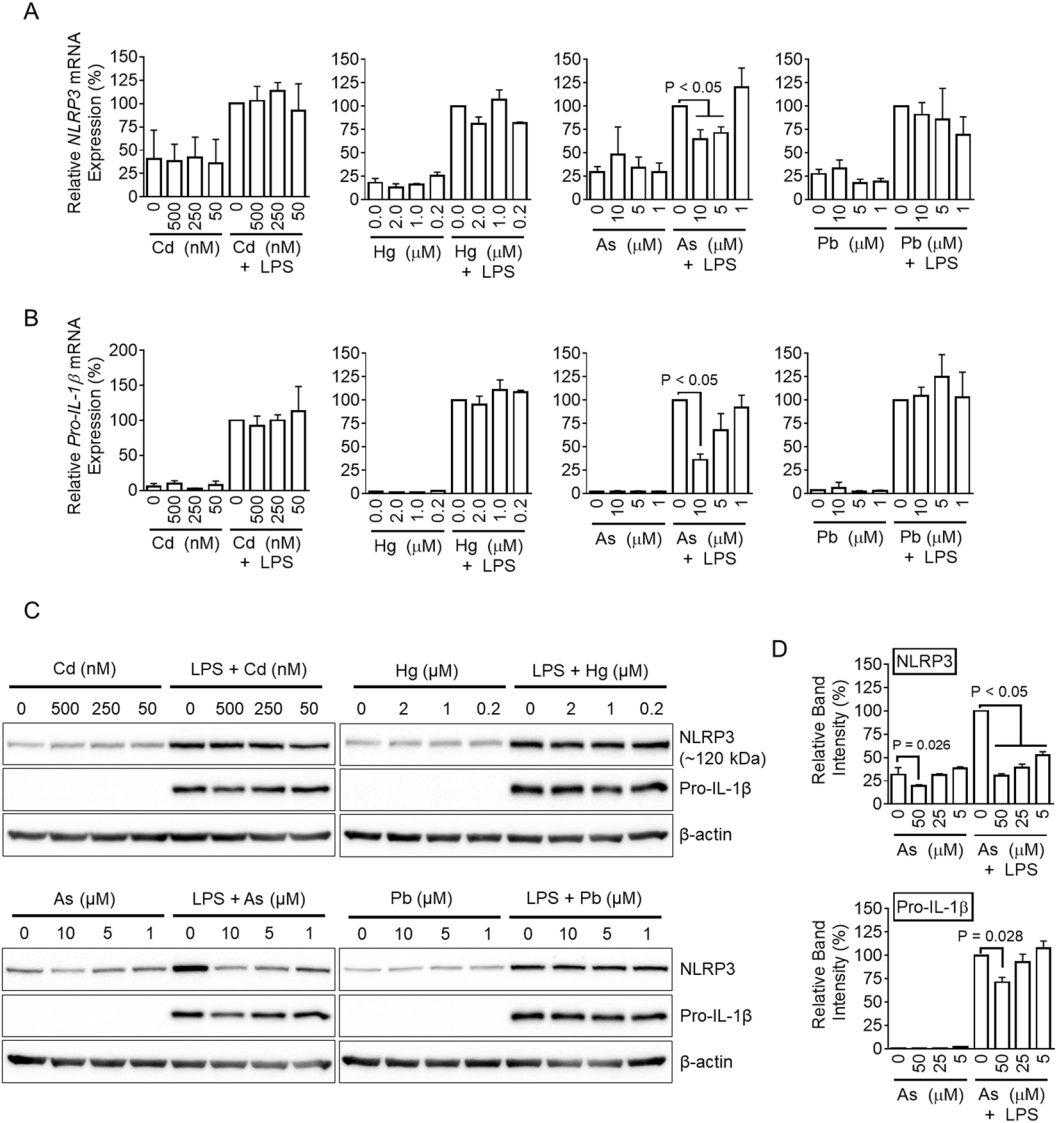
Effects of heavy metals on the priming step. BMDMs were treated with cadmium (Cd), mercury (Hg), arsenic (As), or lead (Pb) with/without LPS (10 ng/ml) for 3 h. The mRNA expression levels of *NLRP3* (**A**) and the pro-form of *IL-1β* (*pro-IL-1β*, **B**) mRNAs were detected by real-time qPCR. (**C**) Protein levels of NLRP3 and pro-IL-1β were detected by immunoblotting. (**D**) Band densities of NLRP3 and pro-IL-1β proteins in response to arsenic treatment are represented as bar graphs. All immunoblot data shown are representative of at least three independent experiments. Bar graph presents the mean ± SD with at least two independent experiments.

For NLRP3 inflammasome activation, macrophages require a priming step, during which cells produce IL-1β precursor and NLRP3 itself through toll-like receptor (TLR) signaling such as TLR4-LPS^32–35^. To elucidate the effects of heavy metals on the priming step of inflammasome activation, BMDMs were treated with heavy metals in the presence of LPS, and the transcriptional and translational levels of NLRP3 and pro-IL-1β were measured. As shown in Fig. 3A and Supplemental Fig. 4, LPS treatment increased expression of *NLRP3* transcription, as expected. Arsenic treatment inhibited LPS-mediated *NLRP3* gene up-regulation, whereas other heavy metals did not. In addition, *pro-IL-1β* mRNA transcription was induced by LPS treatment, and up-regulation was interrupted by arsenic (Fig. 3B). Similar to the transcription data, arsenic attenuated the protein levels of NLRP3 and pro-IL-1β in response to LPS treatment, whereas other heavy metals did not (Fig. 3C,D). Taken together, arsenic not only disrupted activation of inflammasome but also interrupted the priming step of inflammasome activation.

We next investigated whether or not heavy metals could alter expression of other inflammatory cytokines such as IL-1α, TNFα, IL-6, and IL-10 (Supplemental Figs 4 and 5). None of the heavy metals up-regulated cytokines in macrophages. However, arsenic blocked up-regulation of all cytokine gene expression in response to LPS. Interestingly, mercury only blocked *IL-6* and *IL-10* gene expression and did not alter *IL-1β* and *TNFα* mRNA levels. Cadmium and lead did not alter cytokine expression. Taken together, arsenic significantly interrupted both production and maturation of inflammatory cytokines. In addition, mercury partially disrupted cytokine production and maturation.

### Mercury and arsenic inhibit Asc pyroptosome formation, gasdermin D cleavage, and IL-18 maturation resulting from inflammasome activation

**Figure 4:**
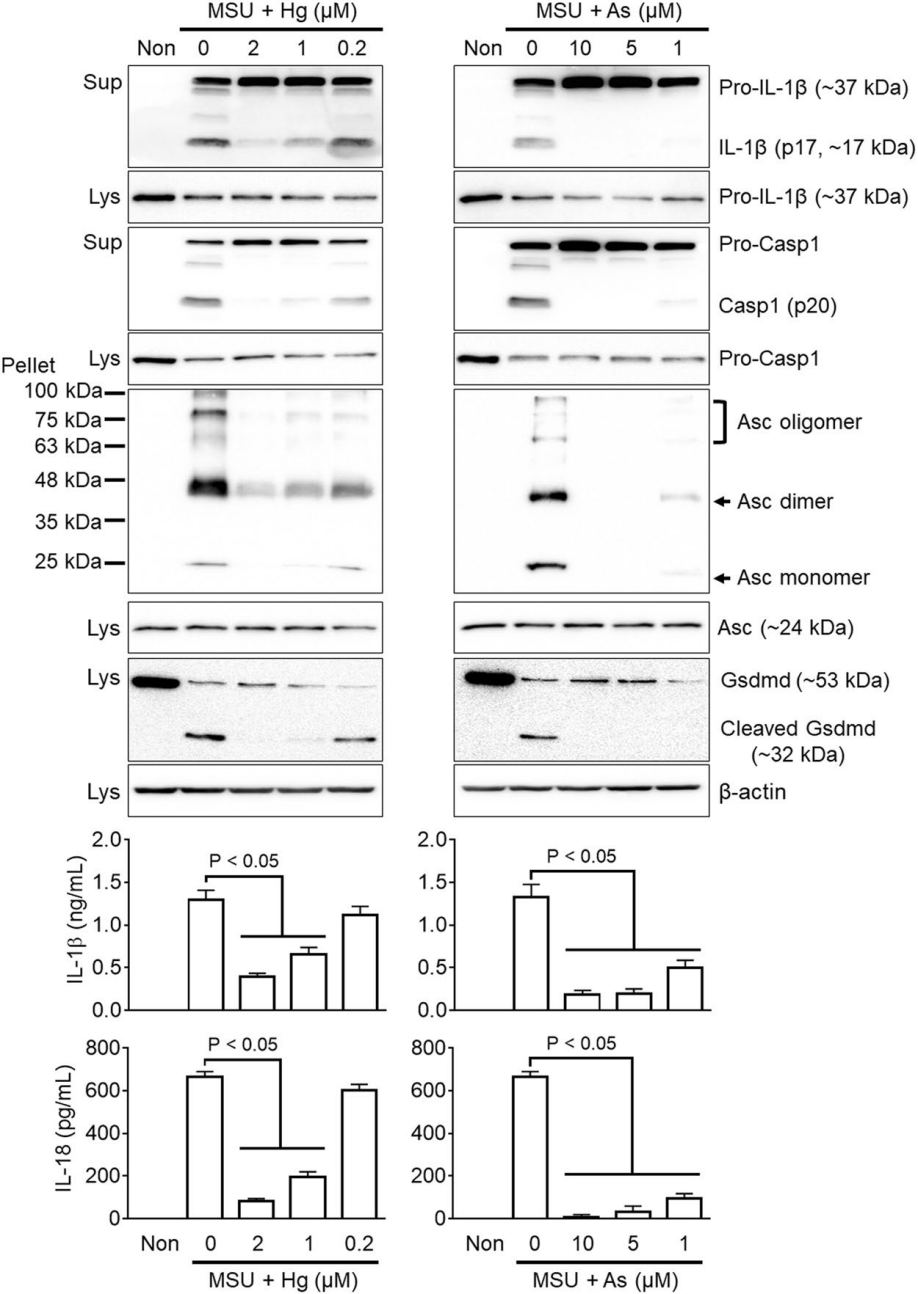
Effects of mercury and arsenic on Asc oligomerization and IL-18 secretion resulting from NLRP3 inflammasome activation. BMDMs were primed with LPS and then treated with MSU in the presence of mercury (Hg, left side) or arsenic (As, right side). Secretion of IL-1β (p17) and Casp1 (p20), Asc pyroptosome formation, and gasdermin D (Gsdmd) cleavage were elucidated by immunoblotting. In addition, release of IL-1β and IL-18 was detected by ELISA. All immunoblot data shown are representative of at least three independent experiments. Bar graph presents the mean ± SD with at least two independent experiments.

To evaluate the upstream and downstream effects of mercury and arsenic on IL-1β maturation and secretion during NLRP3 inflammasome activation, we treated LPS-primed BMDMs with MSU with/without mercury or arsenic and observed formation of Asc pyroptosome and cleavage of gasdermin D (Gsdmd) using immunoblotting (Fig. 4). We also analyzed maturation of IL-1β by immunoblotting as well as secretion of IL-18 using ELISA. Both mercury and arsenic attenuated formation of Asc oligomerization and cleavage of gasdermin D in response to MSU in a dose-dependent manner. In addition, mercury and arsenic attenuated IL-1β maturation and IL-18 secretion. Taken together, mercury and arsenic interrupted Asc pyroptosome formation upstream of caspase-1 cleavage as well as inhibited IL-18 secretion and gasdermin D cleavage.

### Mercury blocks production of mitochondrial reactive oxygen species (ROS) while arsenic interrupts signaling of promyelocytic leukemia protein (PML)-protein phosphatase 2 (PP2a) for inhibition of NLRP3 inflammasome activation

**Figure 5:**
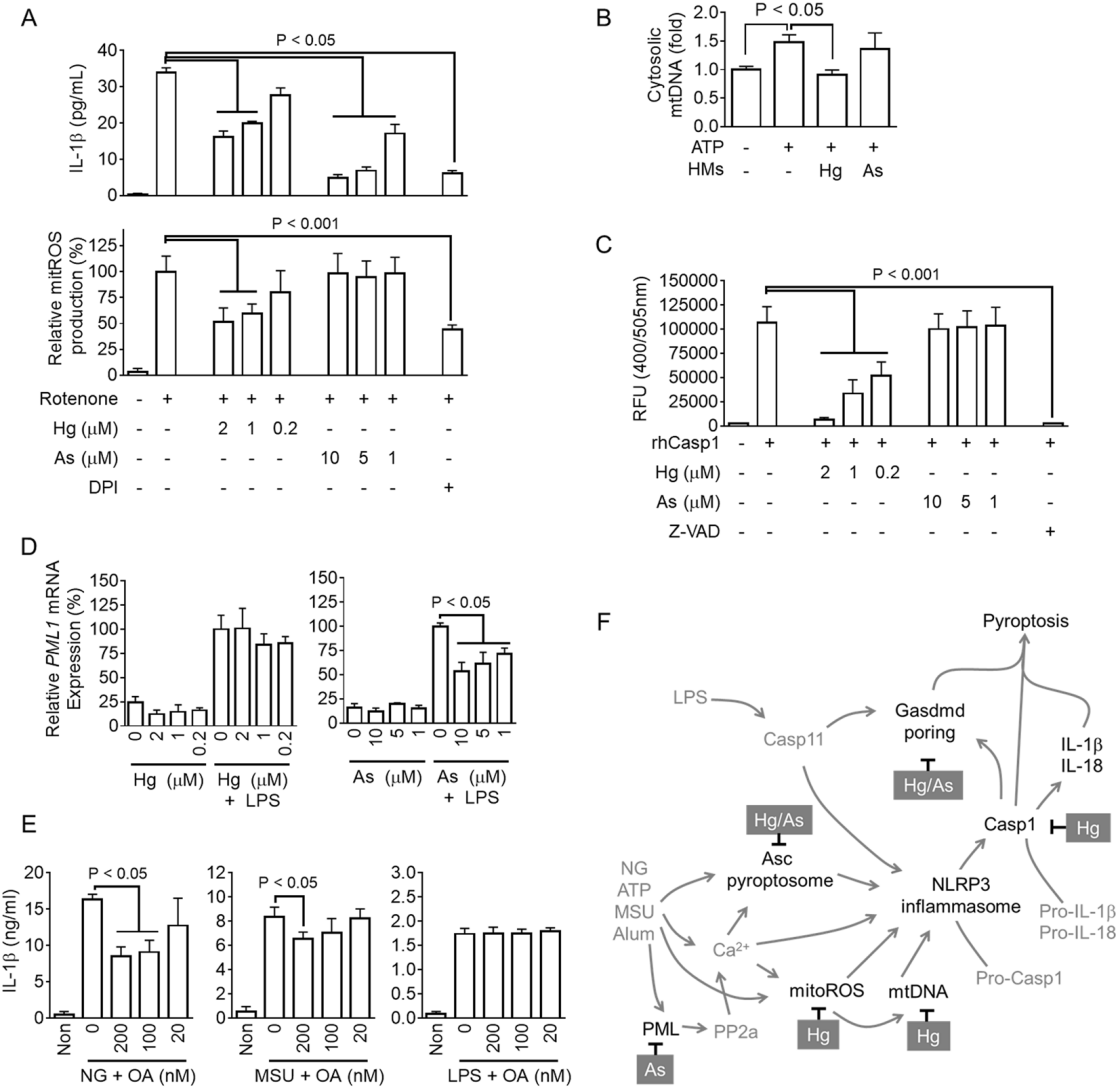
Effects of mercury and arsenic on mitochondrial ROS production, mitochondrial DNA release, caspase-1 activity, and *PML1* gene expression. (**A**) BMDMs were treated with rotenone (160 μM) for 6 h in the presence of mercury (Hg), arsenic (As), or DPI (ROS scavenger). IL-1β secretion was measured by ELISA, and the production of mitochondrial ROS (mitROS) was detected by mitoSOX^TM^. (**B**) LPS-primed BMDMs were treated with ATP (5 mM) and rotenone (5 μM) with/without Hg or As, after which cytosolic release of mitochondrial DNA (mtDNA, cytochrome c oxygenase 1/18r DNA) was analyzed. (**C**) Activity of recombinant human caspase-1 (rhCasp1) was measured in the presence of Hg, As, or Z-VAD-FMK (Z-VAD, pan-caspase inhibitor). (**D**) BMDMs were treated with/without LPS or heavy metals, and expression of PML1 mRNA was quantitated with real-time PCR. (**E**) LPS-primed BMDMs were stimulated for NLRP3 inflammasome activation by treatment with NG or MSU or LPS transfection in the presence of okadaic acid (OA, PP2a inhibitor) as indicated. IL-1β secretion was measured by ELISA. (**F**) Summary of anti-NLRP3 inflammasome properties of Hg and As. Bar graph presents the mean ± SD with at least two independent experiments. RFU, relative fluorescence unit.

To determine the intracellular mechanism responsible for the effects of mercury and arsenic on anti-NLRP3 inflammasome, we adopted rotenone, which blocks mitochondrial respiration resulting in ROS production, to induce NLRP3 inflammasome activation^33,36^. As shown in Fig. 5A, LPS-primed BMDMs induced IL-1β secretion resulting from rotenone-mediated NLRP3 inflammasome activation, and IL-1β secretion was attenuated by diphenyleneiodonium (DPI) treatment, an inhibitor of ROS production. Mercury treatment inhibited both IL-1β and mitochondrial ROS (mitROS) production, implying that mercury attenuated NLRP3 inflammasome activation via blockage of mitROS production. Moreover, we observed the effects of mercury and arsenic on cytosolic translocation of mitochondrial DNA (mtDNA), which is released by mitROS and activates NLRP3 inflammasome^37,38^. As shown in Fig. 5B, mercury inhibited cytosolic mtDNA release in response to ATP and rotenone treatment while arsenic did not. In addition, activity of recombinant human caspase-1 was analyzed in the presence of mercury or arsenic (Fig. 5C). Mercury inhibited caspase-1 activity while arsenic did not. Thus, mercury inhibited NLRP3 inflammasome-mediated caspase-1 activation by interrupting mitROS production and mtDNA release.

Based on the literature, we focused on PML-PP2a signaling since PML-deficient macrophages show a defect in NLRP3 inflammasome activation, and arsenic degrades PML protein in THP-1 cells^39^. PML activates PP2a to release Ca^2+^ from the ER, resulting in direct and/or indirect activation of NLRP3 inflammasome^19,36,39^. As shown in Fig. 5D and Supplemental Fig. 4, arsenic down-regulated expression of *PML1* mRNA while mercury did not. In addition, okadaic acid (OA), an inhibitor of PP2a, selectively attenuated IL-1β secretion in response to NLRP3 triggers. Taken together, arsenic inhibited NLRP3 inflammasome activation through interruption of PML-PP2a signaling, similar to a previous finding^39^.

## Discussion

In this study, we elucidated the effects of heavy metals, cadmium, mercury, arsenic, and lead, on well-characterized inflammasomes, NLRP3, NLRC4, and AIM2 in macrophages. Mercury and arsenic attenuated secretion of IL-1β, IL-18, and caspase-1 resulting from NLRP3 inflammasome activation while cadmium and lead did not. Mercury and arsenic also inhibited non-canonical NLRP3 inflammasome activation but not NLRC4 and AIM2 inflammasome activation. As a non-canonical inflammasome trigger, LPS-mediated serum IL-β secretion was blocked by mercury or arsenic treatment in mice. Inhibition of the canonical and non-canonical NLRP3 inflammasomes by mercury and arsenic was identical in both human and mouse macrophages. Arsenic not only attenuated the activation step of inflammasomes but also interrupted the priming step. As shown in Fig. 5F, mercury attenuated the production of mitochondrial ROS, and cytosolic release of mitochondrial DNA leading to NLRP3 inflammasome activation. Additionally, mercury inhibited capase-1 activity. On the other hand, arsenic down-regulated *PML1* gene expression, resulting in interruption of PML-PP2a signaling. Both mercury and arsenic interrupted formation of Asc pyroptosome and cleavage of gasdermin D. In conclusion, mercury and arsenic selectively inhibited both canonical and non-canonical NLRP3 inflammasome activation, and mercury or arsenic poisoning might disrupt the normal inflammatory response mediated by NLRP3 inflammasomes.

The inflammasome, a critical component of the host immune response, recognizes intracellular danger signals and facilities the inflammatory response for activation of casaspe-1 and secretion of IL-1β and IL-18^40^. NLRP3 inflammasome is not a member of the host immune system but induces metabolic, genetic, and degenerative diseases such as cancer^41^. That is, NLRP3 inflammasome acts as a gatekeeper against pathogen infection, although uncontrolled inflammasome can induce disease. Non-canonical inflammasome also recognizes intracellular Gram negative bacteria and activates caspase-11, resulting in cytokine maturation and pyroptosis^30^. Although pyroptosis is dependent on caspase-11, maturation of IL-1β and IL-18 is dependent on NLRP3 inflammasome^30^. IL-1β, the final product of inflammasome activation, induces expression of adhesion proteins and chemokines in immune cells for recruitment to sites of inflammation and activation^42^. In addition, IL-18 regulates IFNγ and IL-17 production from T lymphocytes and controls tissue homeostasis and repair^42,43^. Although these inflammatory cytokines are important to combat invading pathogens, excessive or insufficient secretion of IL-1β and IL-18 might induce host diseases^44^. In this study, we reported that mercury and arsenic disrupted IL-1β and IL-18 secretions though inhibition of NLRP3 and non-canonical inflammasome activation. This disruption might cause fatal outcomes during pathogen infection.

Mercury is regarded as immunotoxicant since it induces autoimmune disease, over-production of auto-antibodies, and lupus-like symptoms in animal models^45^. In human and mouse macrophages, mercury enhanced expression of IL-1β, TNFα, IL-4, IL-6, IL-17, and INF-γ genes but reduced transcription of IL-1Ra and IL-10 in response to LPS^46,47^. However, mercury blocked LPS-mediated nitric oxide (NO) production through inhibition of NF-κB signaling^47^. In animal studies, mercury administration induced expression of TNFα and IL-6 but not IL-1β^48^. So far, the effect of mercury on expression of inflammatory cytokines was found to be inconsistent. In the current study, mercury alone did not induce any cytokine expression. However, mercury inhibited gene expression of IL-6 and IL-10 in response to LPS, whereas it did not alter IL-1β or TNFα expression. In addition, mercury selectively inhibited NLRP3 and non-canonical inflammasome activation.

The role of arsenic in innate immune cells has been progressively studied, although T lymphocytes have been more studied than monocytes or macrophages. Arsenic exposure was been shown to down-regulate gene expression of IL-1β in human and animal cells via inhibition of TLR/IL-1 receptor and NF-κB signaling^6^. Other cytokines, including TNFα, INFγ, IL-2, IL-6, IL-8, IL-10, and IL-17A, are also interrupted by arsenic^6,49,50^. In macrophages, arsenic inhibits phagocytosis and NO production for digestion of exogenous antigens but induces apoptosis^51^. Arsenic exposure was shown to reduce surface markers of macrophages while enhancing monocytic markers in circulating monocytes, resulting in rapid cell rounding and subsequent loss of adhesion^52^. Arsenic treatment was found to alter polarization of macrophages to M2 status, in which macrophages show increased IL-10 and TGF-β levels^53^. Thus, arsenic exposure blocks inflammatory responses of macrophages, which recognize and eliminate harmful signals such as endogenous and exogenous antigens through inhibition of pro-inflammatory cytokines. In addition, we report here that arsenic also interrupted activation of inflammasomes, resulting in maturation of IL-1β and IL-18.

Interestingly, arsenic, especially arsenic trioxide, shows beneficial effects on patients having acute promyelocytic leukemia, multiple myeloma, and myelodysplastic syndrome^54^. Arsenic trioxide also was shown to ameliorate symptoms of systemic lupus erythematosus (SLE) in a mouse model^55^. Inflammasomes might be involved in the therapeutic mechanism of arsenic trioxide in the above diseases. Promyelocytic leukemia (PML) proteins, which fuse with retinoic acid receptor alpha and induce tumors such as acute promyelocytic leukemia, were shown to be down-regulated by arsenic trioxide, and PML proteins are tightly involved in NLRP3 inflammasome activation^39^. IL-1 inhibitory therapy by IL-1 receptor antagonist (IL-1Ra) ameliorates the progression of active multiple myeloma^44^, and NLRP3 inflammasome was reported as a hallmark of myelodysplastic syndrome^56^. In addition, macrophages derived from SLE patients easily activate NLRP3 inflammasome, and this inflammasome was reported to cause lupus nephritis^57^. Thus, the inhibitory effect of arsenic on NLRP3 inflammasome could be suggested as the mechanism behind the effect of arsenic trioxide on disease.

## Materials and Methods

### Preparation of macrophages and cell culturing

BMDMs were differentiated from bone marrow progenitors isolated from the tibia and femur bones of C57BL/6 mice (6- to 12-weeks-old; Narabio Co., Seoul, Republic of Korea) in L929 cell-conditioned medium (LCCM) containing macrophage colony-stimulating factor. In brief, progenitors were plated in non-tissue culture-treated dishes (SPL Life Science Co., Gyeonggi-do, Republic of Korea) and incubated at 37 °C in a 5% CO_2_ atmosphere for 7 days. The cells were supplied with RPMI 1640 medium (RPMI-A, Capricorn Scientific GmbH, Edsdorfergrund, Germany) containing 10% fetal bovine serum (FBS; FBS-22A, Capricorn Scientific GmbH), 30% LCCM, and antibiotics (100 U/ml of penicillin and 100 µg/ml of streptomycin; CA005, GenDEPOT, Inc., Barker, TX, USA).

### Cell treatment

BMDMs were seeded in 12-well plates (1.0 × 10^6^ cells per well, SPL Life Science Co.) and primed with 1 μg/ml of lipopolysaccharide (LPS; L4130, Sigma–Aldrich Co., MO,USA) in RPMI 1640 medium containing 10% FBS and antibiotics for 3 h. After LPS priming, cells were subjected to the activation steps as follows. To activate NLRP3 inflammasome, cells were treated with RPMI 1640 medium (350 μl/well) without FBS or antibiotics in the presence of nigericin (NG, 40 μM; 4312, Tocris Bioscience, Bristol, UK) for 1 h, monosodium urate crystals (MSU, 400 μg/ml; U2875, Sigma–Aldrich Co.) which were prepared in according to a previous study^58^ for 3 h, aluminium potassium sulfate (Alum, 200 μg/ml; 039–4404, Daejung Chemicals & Materials Co., Daejeon, Republic of Korea) for 3 h, or rotenone (160 μM; sc-203242, Santa Cruz Biotechnology, Dallas, TX, USA) for 6 h. For non-canonical inflammasome activation, cells were transfected by LPS (15 μg/ml, Sigma-Aldrich Co) with Lipofectamine 2000 (10 μl/ml, Invitrogen) and incubated for 6 h or infected by *Escherichia coli* (MOI 10, DH5α, Invitrogen, CA, USA) for 3 h. Heavy metals, cadmium acetate dihydreate (CAS No. 5743-04-04, Cat. No. 2616-4140), mercury(II) chloride (CAS No. 7487-94-7, Cat. No. 5542-4425), arsenic(III) oxide (arsenic trioxide, CAS No. 1327-53-3, Cat. No. 5076-1405), lead(II) acetate trihydrate (CAS No. 6080-56-4, Cat. No. 5075-4405), or lead(II) chloride (CAS No. 7758-95-4, Cat. No. 5076-1405), were purchased from Daejung Chemicals & Materials Co. and co-treated with the above inflammasome triggers. Okadaic acid (Cayman Chemical, Ann Arbor, MI, USA) were treated with inflammasome triggers. To analyze the effects of heavy metals on the priming step and cytokine expression, BMDMs (2.0 × 10^6^ cells per well) seeded in 6-well plates (SPL life science Co.) were treated with heavy metals as indicated with/without LPS (10 ng/ml) for 3 h.

### Animal study

Female C57BL/6 mice (8-weeks-old; Narabio Co.) were provided standard sterile chow diet and water *ad libitum* and allowed to adjust to the environment for 1 week under a 12 h light/dark cycle at 24 °C. Mice (n = 5 per group, total n = 30) were intraperitoneally (ip) administrated with PBS (200 μl), mercury(II) chloride (12 μg/mouse, 0.6 mg/Kg) or arsenic(III) oxide (40 μg/mouse, 2 mg/Kg) 30 min after ip injection of PBS (200 μl) or LPS (100 μg/mouse). After an additional 5.5 h, mice were euthanized by CO_2_ inhalation, and blood was collected for cytokine assay. All animal experiments were carried out in accordance with the National Institutes of Health Guide for the Care and Use of Laboratory Animals and approved by the Institutional Animal Care and Use Committee of Kangwon National University (IACUC; approval no. KW-170110-1 and KW-180306-2).

### Western blotting sample preparation

For inflammasome activation, cellular supernatant (Sup, >250 μl) was collected, and the remaining cells were lysed with 100 μl of mild lysis buffer (150 mM NaCl, 1% Triton X-100, 50 mM Tri-base, pH 8.0) containing proteinase inhibitor cocktail (M250-1, AMRESCO LLC, Solon, OH, USA). The lysate (Lys) was harvested into a new tube by centrifugation at 15,000 rcf for 5 min. The remaining pellet was washed two times with PBS and then re-suspended and cross-linked with 2 mM suberic acid bis (S1885, Sigma-Aldrich Co.) for 1 h, followed by centrifugation at 15,000 rcf for 5 min. The cross-linked pellets (Pellet) were re-suspended in 50 μL of 2 X loading dye buffer (116 mM Tris, 3.4% SDS, 12% glycerol, 200 mM DTT, 0.003% bromo phenol blue). The Sup, Lys, and Pellet were subjected to Western blot assay.

### Western blot analysis

Sup, Lys, and Pellet samples were separated by SDS-PAGE (10% or 16%) using running buffer (25 mM Tris, 192 mM glycine, 0.1% SDS, pH 8.3) and Mini-PROTEAN® Tetra Handcast Systems (BIO-RAD, Hercules, CA, USA) and transferred onto a polyvinylidene difluoride membrane (PVDF; 10849A, Pall Co., Port Washington, NY, USA) using transfer buffer (25 mM Tris, 192 mM glycine, 10% methanol, pH 8.3) and Criterion^TM^ Blotter (BIO-RAD). The membrane was probed with primary antibodies against anti-mouse IL-1β antibody (AF-401-NA, R&D Systems, Minneapolis, MN, USA), anti-Caspase-1(p20) antibody (AG-20B-0042-C100, AdipoGen^®^ Co., San Diego, CA, USA), anti-Asc antibody (sc-22514, Santa Cruz Biotechnology), anti-NLRP3 antibody (AG-20B-0014-C100, AdipoGen^®^ Co.), anti-GSDMD antibody (ab209845, Abcam plc., Cambridge, MA, USA), or anti-Actin antibody (sc-1615, Santa Cruz Biotechnology) overnight at 4 °C. The membranes were further probed with HRP-conjugated 2^nd^ anti-sera (sc-2020 or sc-2004, Santa Cruz Biotechnology) and visualized using a chemiluminescence detection solution (WESTSAVER STAR, AbFrontier, Seoul, Republic of Korea) and a cooled CCD camera System (AE-9150, EZ-Capture II, ATTO Technology, Tokyo, Japan). Full-length bolts for figures were shown in Supplementary information.

### Detection of IL-1β or IL18 using ELISA

Release of IL-1β or IL-18 were detected using an IL-1β/IL-1F2 Quantikine ELISA Kit (DY201 or DY401, R&D Systems) or mouse IL-18 platinum ELISA (BMS618/3, eBioscience, San Diego, CA, USA). The ELISA plates were readout using a microplate spectrophotometer (Synergy™ H1 Hybrid Multi-Mode Reader, BioTek, Winooski, VT, USA).

### Cytosolic mitochondrial DNA assay

LPS-primed BMDMs (2 × 10^6^ cells per well in 6-well plate) were treated with ATP (5 mM) and rotenone (5 μM) for 1 h, and cytosolic DNA was prepared according to a previous report^37^. Briefly, cells were washed with PBS and administered 100 μl of IGEPAL CA-630 (1%, I8896, Sigma–Aldrich Co.) for lysis. After incubation on ice for 15 min, the cytosolic fraction was isolated by centrifugation at 15,000 rcf for 30 min at 4 °C and subjected to G-spin^TM^ total DNA extraction kit (iNtRON biotechnology, Gyeonggi-do, Republic of Korea) to harvest cytosolic DNA. Copy numbers of cytochrome c oxidase 1 (mitochondrial gene) and 18S rDNA (control) were quantitated with real-time PCR.

### RNA extraction and real-time PCR

Total RNA was extracted using NucleoZOL (MACHEREY-NAGEL GmbH & Co. KG, Postfach, Düren, Germany) and reverse-transcribed into first-strand complementary DNA (cDNA) using random primer (9-mer) and M-MLV reverse transcriptase (Enzynomics Co., Daejeon, Korea) according to the manufacturer’s protocols. Transcripts were quantitated using TOPreal™ qPCR 2X PreMIX (Enzynomics Co.) and the Eco Real-Time PCR system (Illumina, San Diego, CA, USA). Quantitation was normalized with GAPDH. NLRP3 (*Nlrp3*; Genebank ID: NM_145827) 5′-CAG GCG AGA CCT CTG GGA AA-3′ and 5′-CCC AGC AAA CCC ATC CAC TC-3′; IL-1β (*Il1b*; NM_008361) 5′-CCC AAG CAA TAC CCA AAG AA-3′ and 5′-GCT TGT GCT CTG CTT GTG AG-3′; PML1 (*pml1*; NM_178087) 5′-GGA GAA CGA GGA CAG GTT GG-3′ and 5′-TCT CGG TGT CCG AAT CCT CT-3′; GAPDH (*Gapdh*; NM_001289726) 5′-AAC TTT GGC ATT GTG GAA GG-3′ and 5′-ACA CAT TGG GGG TAG GAA CA-3′; 18 s rDNA^37^ 5′-TAG AGG GAC AAG TGG CGT TC′ and 5′-CGC TGA GCC AGT CAG TGT-3′; cytochrome c oxidase 1^37^ 5′-GCC CCA GAT ATA GCA TTC CC-3′ and 5′-GTT CAT CCT GTT CCT GCT CC-3′.

### Mitochondrial ROS and caspase-1 activity assays

BMDMs (1.25 × 10^5^ cells per well) plated in a 96-well black plate (SPL Life Science Co.) were incubated with MitoSOX^TM^ Red mitochondrial superoxide indicator (2.5 μM, M36008, Invitrogen) for 30 min at 37 °C. Cells were treated with rotenone (160 μM; sc-203242, Santa Cruz Biotechnology) in the presence of mercury, arsenic, or diphenyleneiodonium (DPI, 0504, 200 μM, Tocris Bioscience) or for 6 h at 37 °C. For caspase-1 activity, human recombinant caspase-1 (1 unit/rx, BioVision Inc., Milpitas, CA, USA) was incubated with obovatol or a caspase inhibitor (Z-VAD-FMK, 10 μg/ml; APExBIO, Boston, MA, USA) in the presence of caspase-1 substrate, YVAD-pNA, using a caspase-1/ICE Fluorometric Assay kit (BioVision Inc.). The plates were readout using a plate reader (510/580 nm, Synergy™ H1 Hybrid Multi-Mode Reader, BioTek).

### Statistical analyses

Statistical analyses were performed using a t-test (Mann-Whitney test) for the two groups or one-way ANOVA (Tukey’s multiple comparisons test) for multiple groups using GraphPad Prism 6 (GraphPad Software, San Diego CA). P value and fixed factors of the variance analysis are indicated in the figure.

## Electronic supplementary material

Supplementary data
