# Supplementary material for: Mercury and arsenic attenuate canonical and non-canonical NLRP3 inflammasome activation
Source: Sci Rep. 2018 Sep 12;8:13659. doi: 10.1038/s41598-018-31717-7 (PMC6135747; doi:10.1038/s41598-018-31717-7)

Supplementary Information

Mercury and arsenic attenuate canonical and non-canonical NLRP3 inflammasome activation

Huijeong Ahn[1#], Jeongeun Kim[1#], Seung Goo Kang[2], Sung-il Yoon[3], Hyun-Jeong Ko[4], Pyeung-Hyeun Kim[2], Eui-Ju Hong[5], Beum-Soo An[6], Eunsong Lee[1], and Geun-Shik Lee[1]*

[1]College of Veterinary Medicine and Institute of Veterinary Science; [2]Department of Molecular Bioscience, School of Biomedical Science; [3]Division of Biomedical Convergence, College of Biomedical Science; [4]Laboratory of Microbiology and Immunology, College of Pharmacy, Kangwon National University, Chuncheon, Gangwon, 24341, Republic of Korea.

[5]College of Veterinary Medicine and Institute of Veterinary Science, Chungnam National University, Daejeon, 34134, Republic of Korea.

[6]Department of Biomaterial Science, College of Natural Resources and Life Science, Pusan National University, Gyeongsangnam-do, 50612, Republic of Korea

[#]These authors contributed equally to this work.

*Correspondence: Geun-Shik Lee, D. V. M., Ph. D.

Laboratory of Inflammatory Diseases, Department of Physiology, College of Veterinary Medicine, Kangwon National University, Chuncheon, Gangwon, 24341, Republic of Korea.

e-mail: leegeun@kangwon.ac.kr, Tel: +82-33-250-8683, Fax: +82-33-244-2367

Supplemental figure 1.

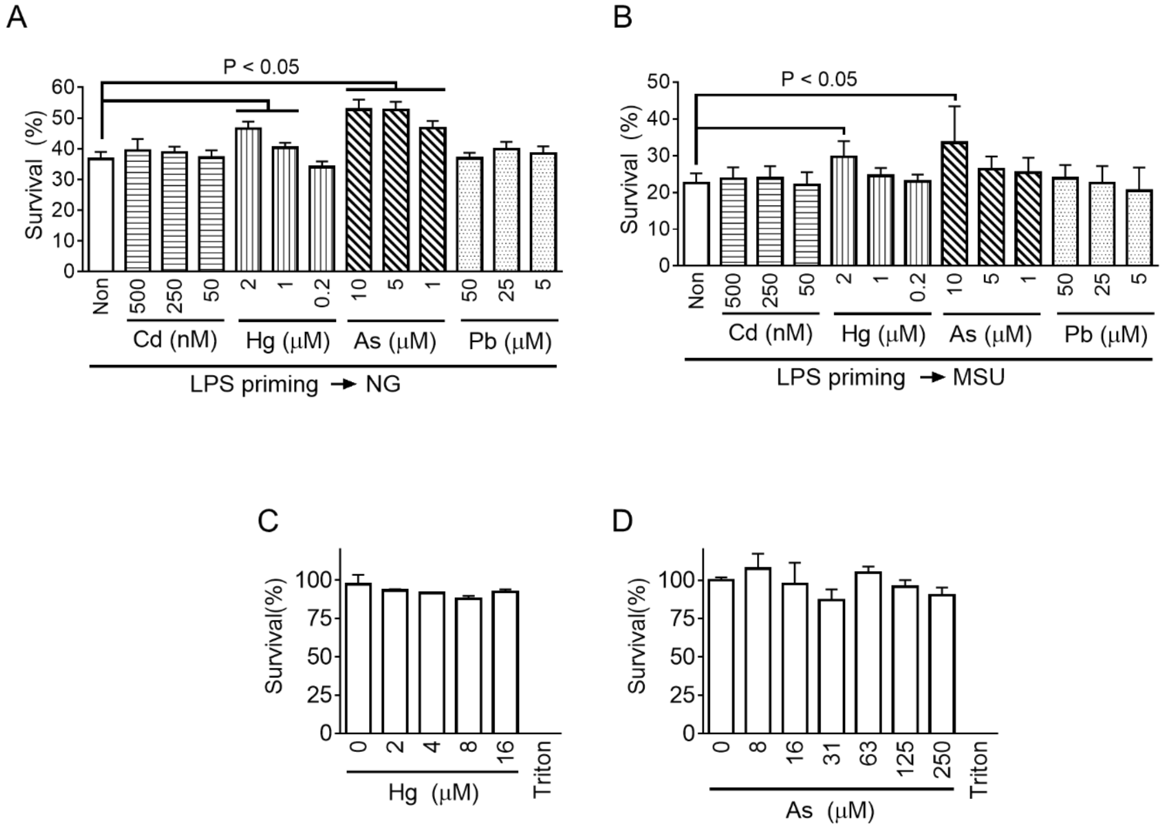

Supplemental figure 1. Inflammasome-dependent cell death and cytotoxicity

BMDMs (50,000 cells / well) were plated in 96-well plates (SPL life science Co.) and primed with LPS (1 μg/ml) for 3 h. LPS-primed BMDMs were treated with NG (**A**; 40 μM, for 1 h) or MSU (**B**; 400 μg/ml, for 3 h) in the presence of heavy metals as indicated. BMDMs were treated with mercury (Hg, **C**) or arsenic (As, **D**) for 6 h. Triton x-100 (0.01%, Triton) was used to obtain complete cell death (0% survival rate) while the non-treated group was set as 100%. Survival rates were measured by EZ-Cytox^{TM} Enhanced cell viability assay kit (Daeilab service co., Seoul, Republic of Korea) was used per the manufacturer’s protocol.

Supplemental figure 2

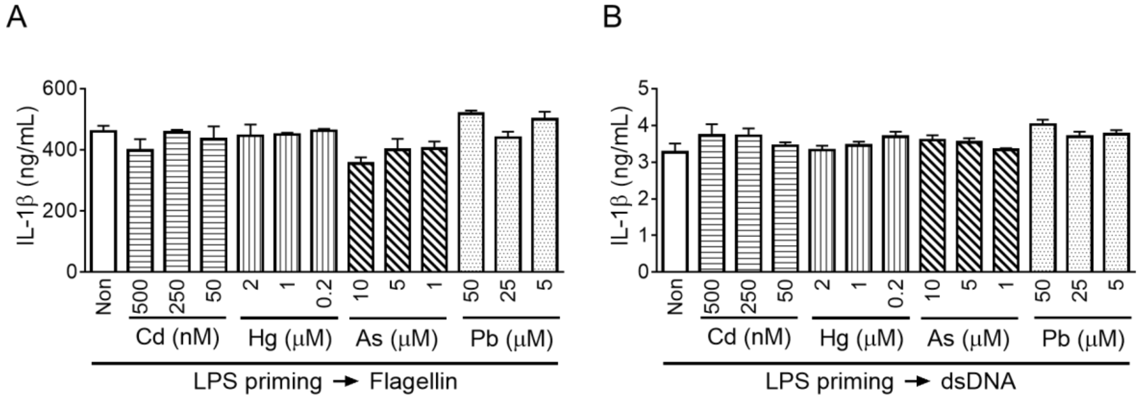

Supplemental figure 2. Effects of heavy metals on NLRC4 and AIM2 inflammasome activation

BMDMs were primed with LPS (1 μg/ml) for 3 h. To activate NLRC4 inflammasome, LPS-primed macrophages were treated with flagellin (**A**; 0.5 μg/ml; tlrl-stfla, InvivoGen, San Diego, CA, USA) with Lipofectamine 2000 (10 μl/ml, Invitrogen, Grand Island, NY, USA) for 1 h. For AIM2 inflammasome activation, LPS-primed cells were transfected with dsDNA (**B;** 1 μg/ml) with jetPRIME^{TM} (2 μl/ml, Polyplus-transfection Inc., Illkirch, France) for 1 h. IL-1β secretion was measured using an IL-1β/IL-1F2 Quantikine ELISA Kit (DY401, R&D Systems). Bar graph presents the mean ± SD.

Supplemental figure 3

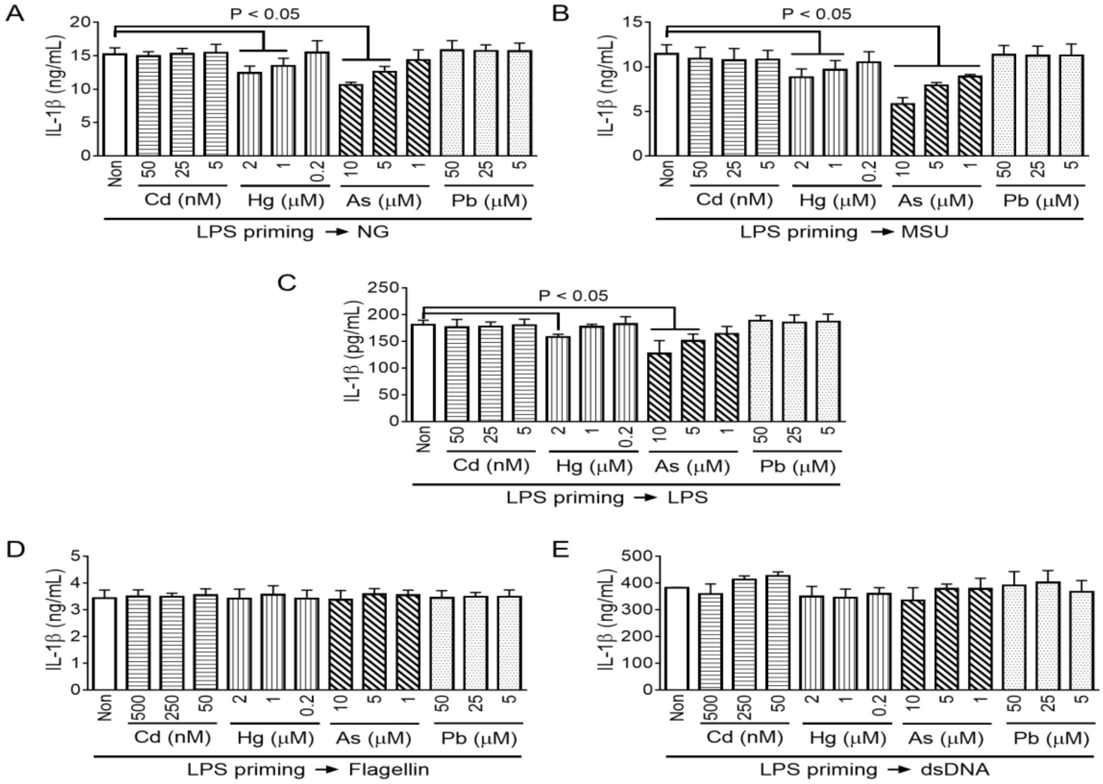

Supplemental figure 3. Effects of heavy metals on NLRP3, NLRC4, and AIM2 inflammasome activation in THP-1 cells.

THP-1 cells were purchased from Korea Cell Line Bank (KCLB No. 40202; Seoul, Republic of Korea) and grown in RPMI 1640 medium containing 10% FBS and antibiotics at 37 °C in a 5% CO_2 atmosphere. THP-1 cells were differentiated into macrophage-like cells by treatment with phorbol 12-myristate 13-acetate (100 nM, PMA; tlrl-pma, InvivoGen, CA, USA) for 72 h. PMA-treated THP-1 cells were primed with LPS (1 μg/ml) for 3 h and then treated with NG (**A**; 40 μM) for 1 h, MSU (**B**; 400 μg/ml) for 3 h, LPS (**C**; 15 μg/ml) with Lipofectamine 2000 (10 μl/ml) for 6 h, flagellin (**D** μg/ml) with Lipofectamine 2000 (10 μl/ml) for 1 h, or dsDNA (**E**; 1 μg/ml) with jetPRIME^{TM} (2 μl/ml) for 1 h in the presence of heavy metals as indicated. IL-1β secretion was measured using an IL-1β/IL-1F2 Quantikine ELISA Kit (DY201, R&D Systems). Bar graph presents the mean ± SD.

Supplemental figure 4

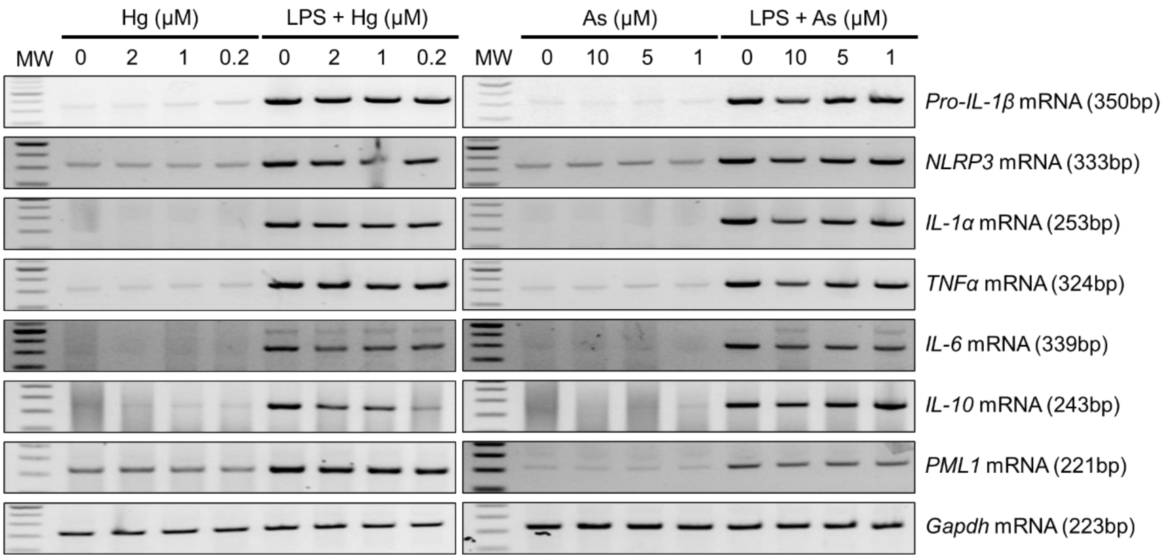

Supplemental figure 4. Effects of mercury or arsenic on mRNA expression of *NLRP3, PML1* and cytokines in BMDMs

BMDMs (2.0 × 10^6 cells per well, 6-well plate) were treated with mercury (Hg) or arsenic (As) with/without LPS (10 ng/ml) for 3 h. Total RNA was extracted using NucleoZOL (MACHEREY-NAGEL GmbH & Co. KG, Postfach, Düren, Germany) and reverse-transcribed into first-strand complementary DNA (cDNA) using an M-MLV cDNA Synthesis kit (Enzynomics, Daejeon, Korea). Transcription was amplified using a SimpliAmp™ Thermal Cycler (Thermo Fisher Scientific Inc. Grand Island, NY, USA) and nTaq polymerase (Enzynomics). PCR products were visualized by agarose gel electrophoresis, ethidium bromide staining, and EZ-Capture™ II (ATTO Technology). IL-1α (*Il1a*; NM_010554) 5′-CCG ACC TCA TTT TCT TCT GG-3′; TNFα (*Tnfa*; NM_013693) 5′-ACG GCA TGG ATC TCA AAG AC-3′ and 5′-GTG GGT GAG GAG CAC GTA GT-3′; and 5′-GTG CAC CCG ACT TTG TTC TT-3′; IL-6 (*Il6*; NM_031168) 5′-CCG GAG AGG AGA CTT CAC AG-3′ and 5′-TCC ACG ATT TCC CAG AGA AC-3′; IL-10 (*Il10*; NM_010548) 5′-TGC TAT GCT GCC TGC TCT TA-3′ and 5′-TCA TTT CCG ATA AGG CTT GG-3′;

Supplemental figure 5

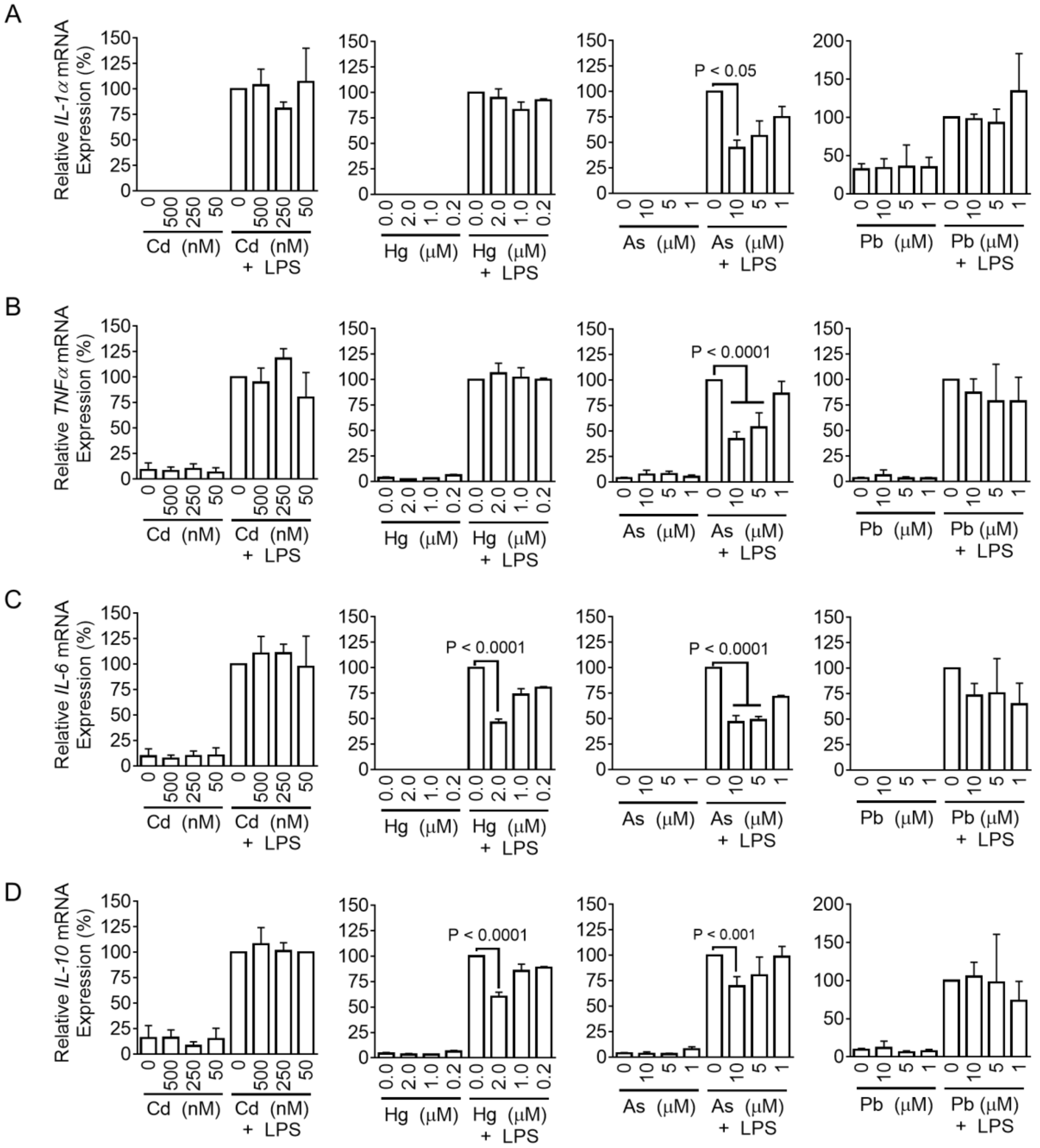

Supplemental figure 5. Effects of heavy metal on cytokine expression

BMDMs were treated with cadmium (Cd), mercury (Hg), arsenic (As), or lead (Pb) with/without LPS (10 ng/ml) for 3h. The mRNA expression levels of *IL-1α* (**A**), *TNFα* (**B**), *IL-6* (**C**), and *IL-10* (**D**) were analyzed by qRT-PCR. Bar graph presents the mean ± SD.

Full-length blots of Figure 1C

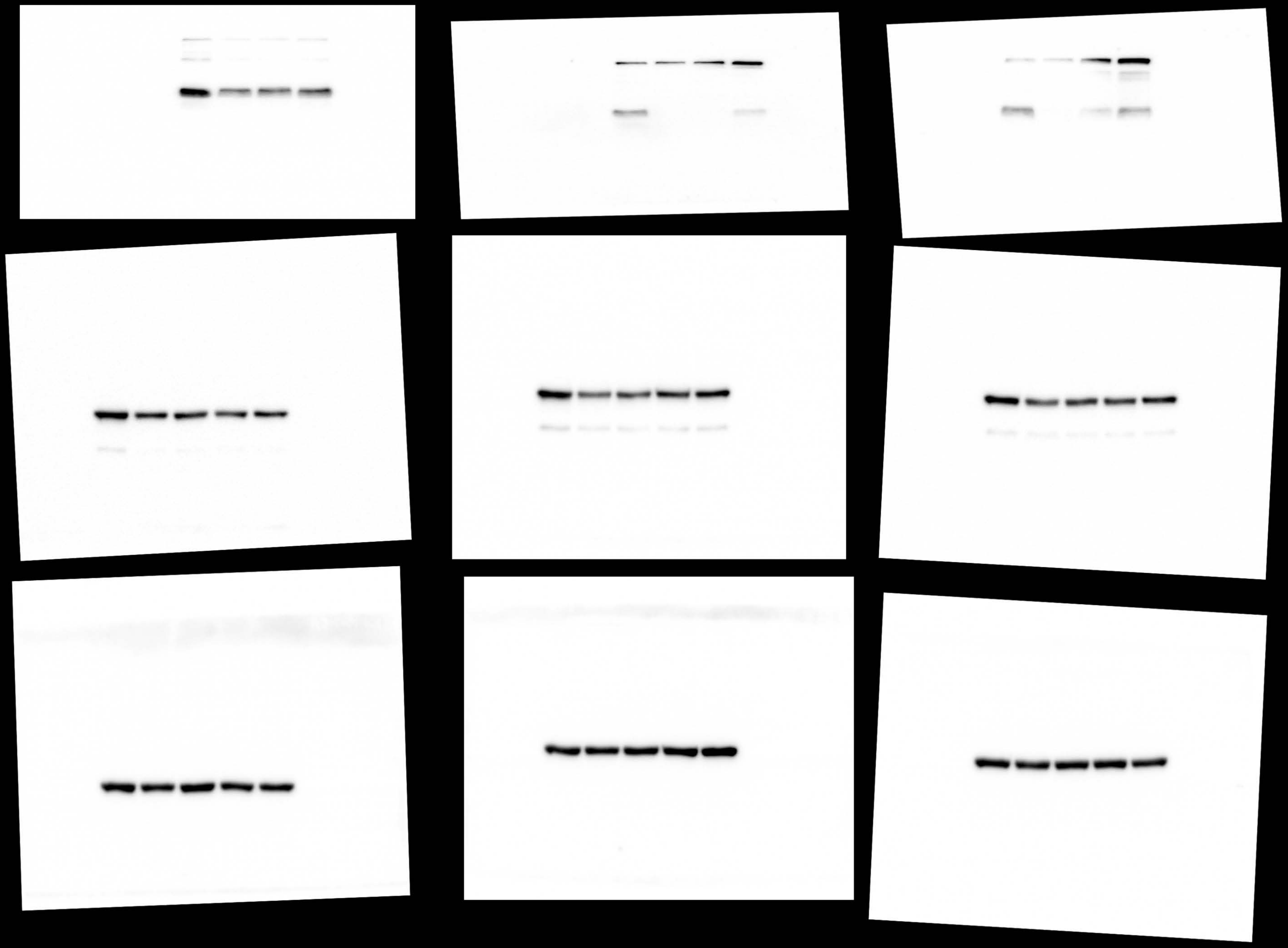

Full-length blots of Figure 1D

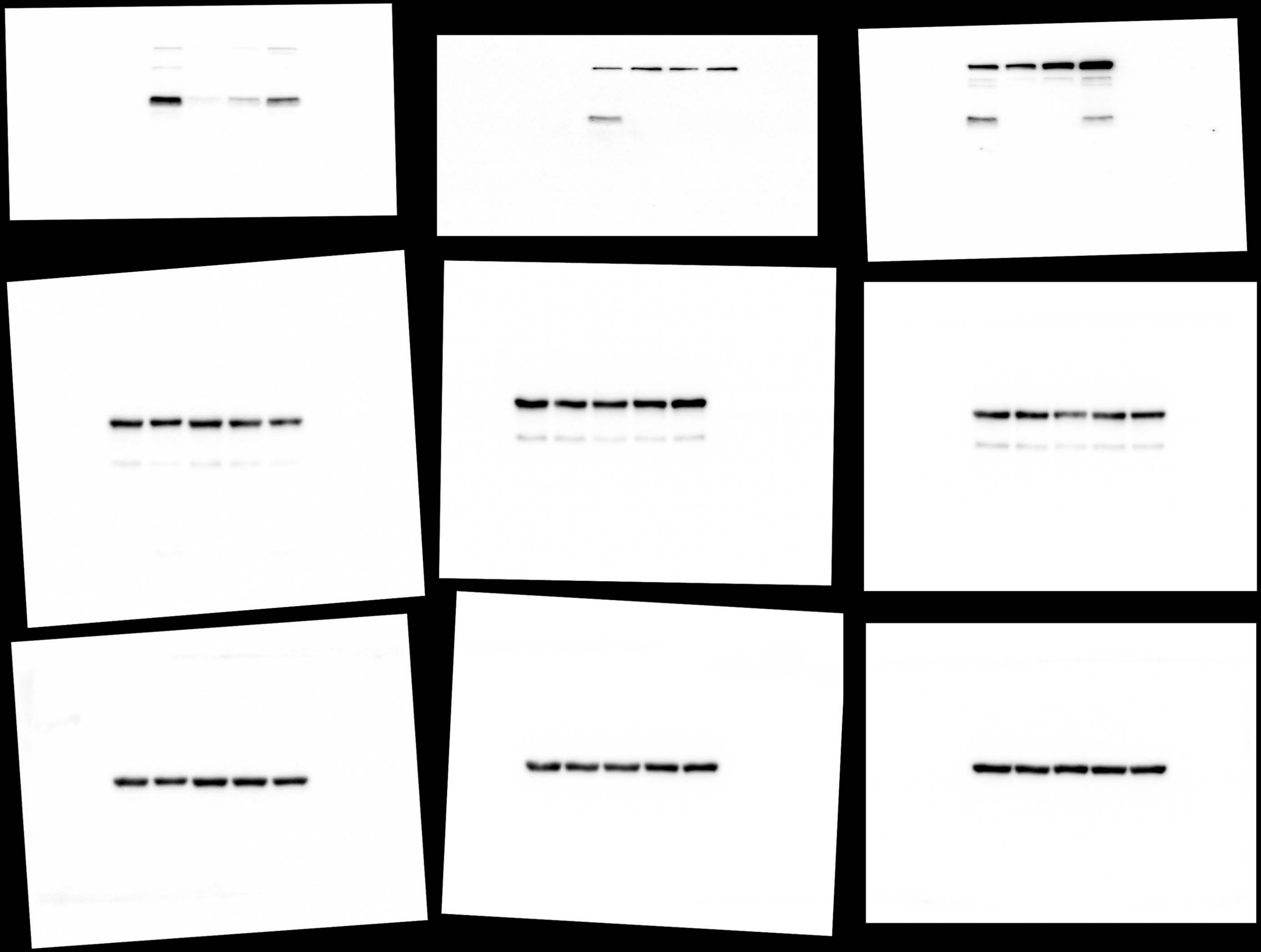

Full-length blots of Figure 2B

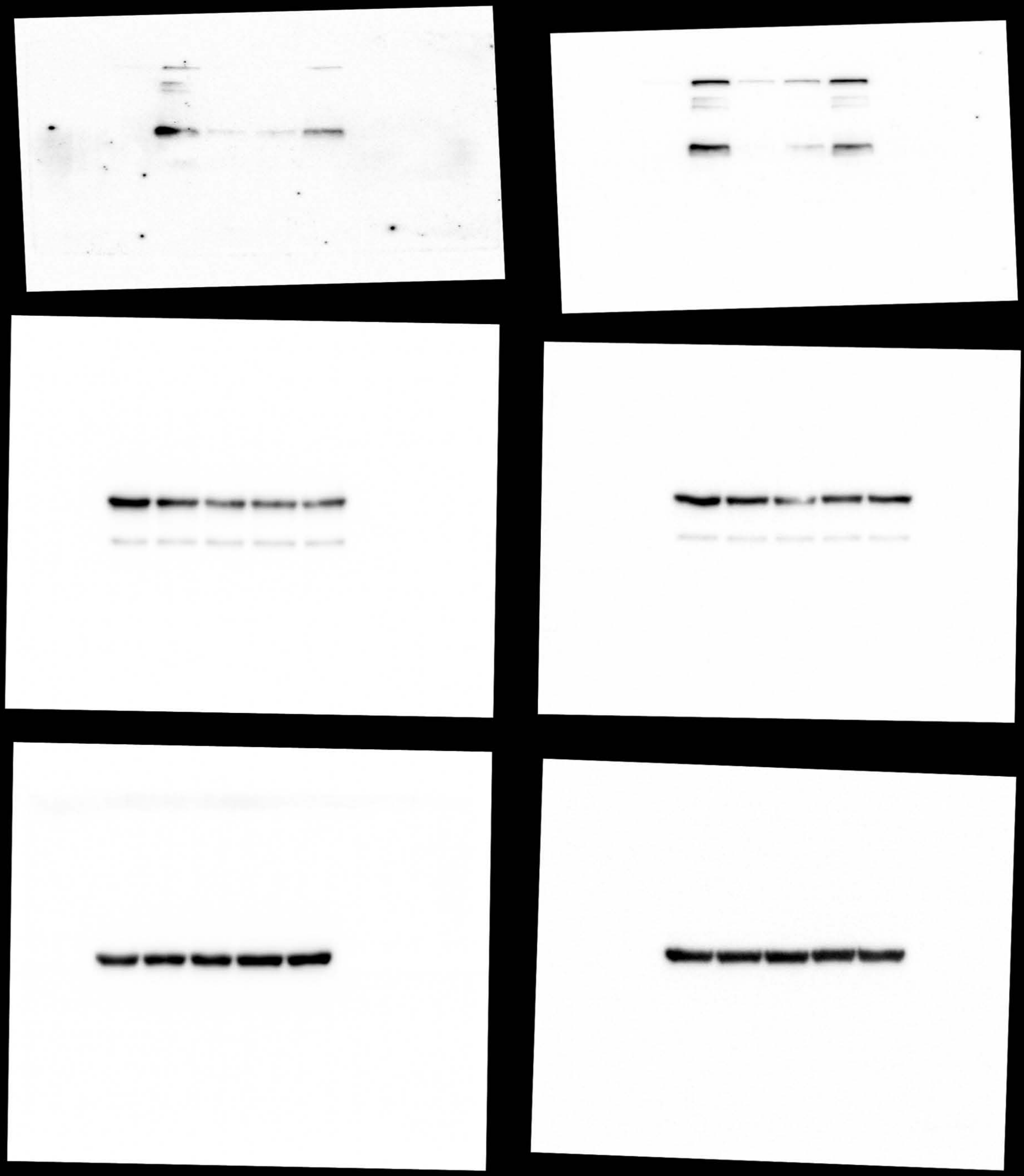

Full-length blots of Figure 2C

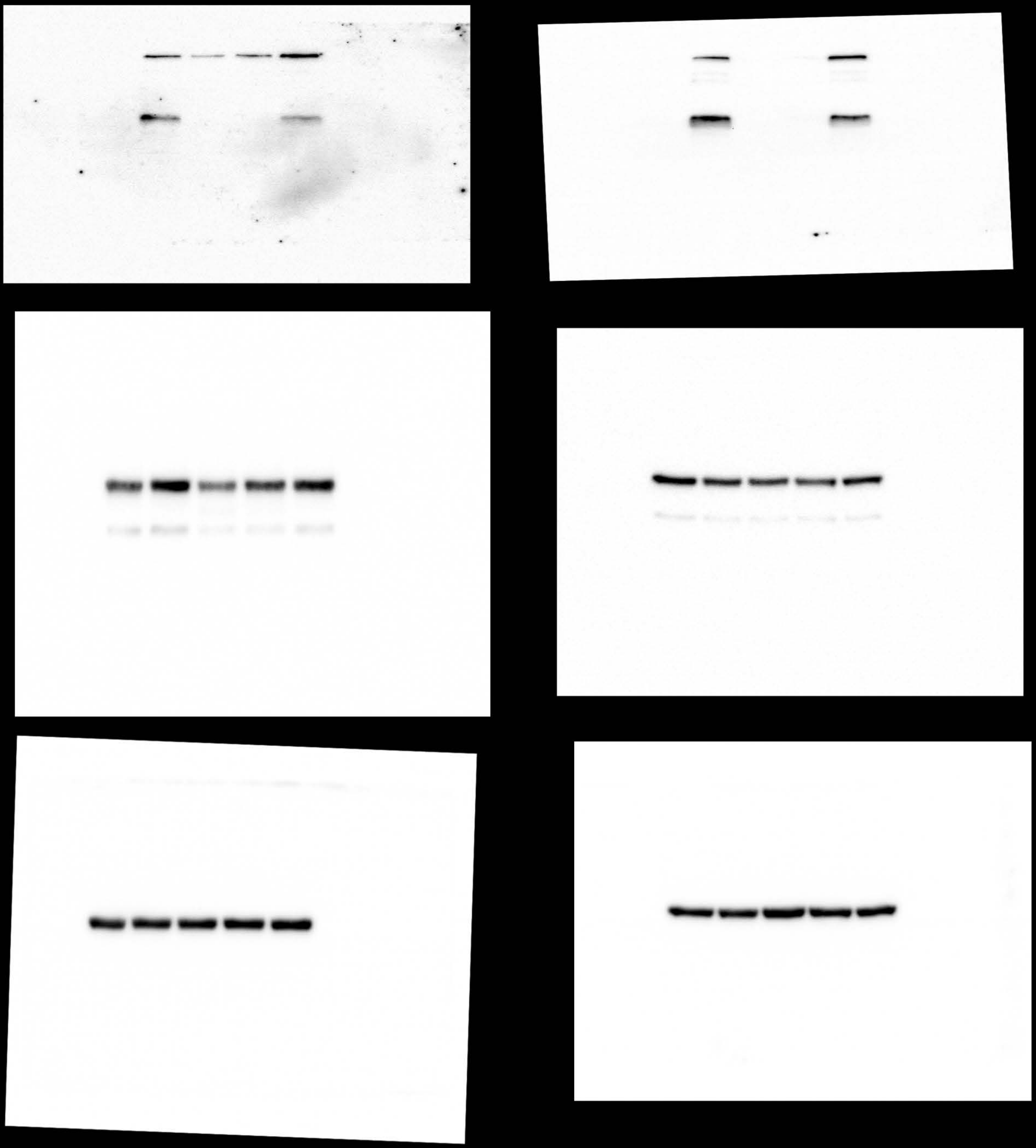

Full-length blots of Figure 3C

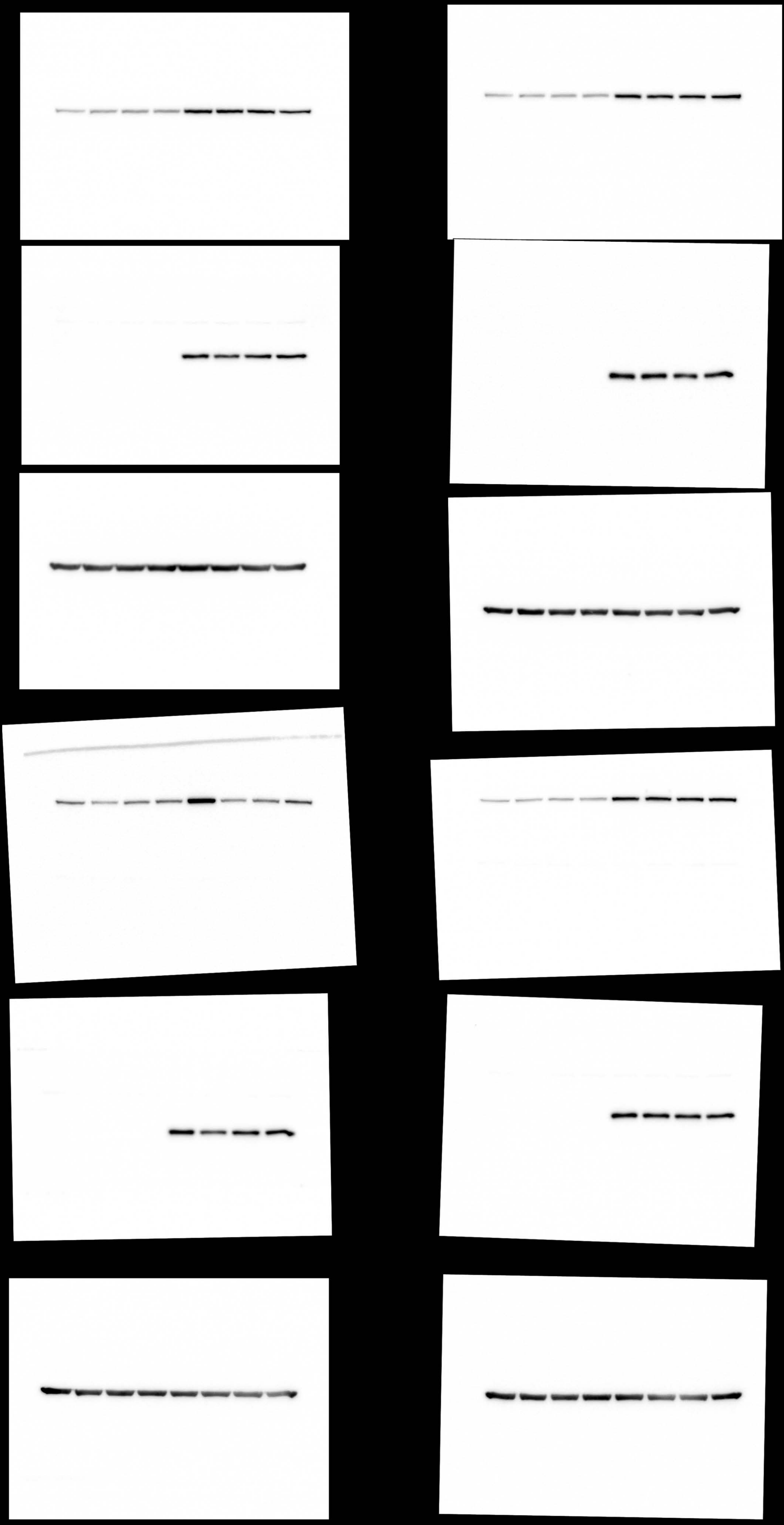

Full-length blots of Figure 4

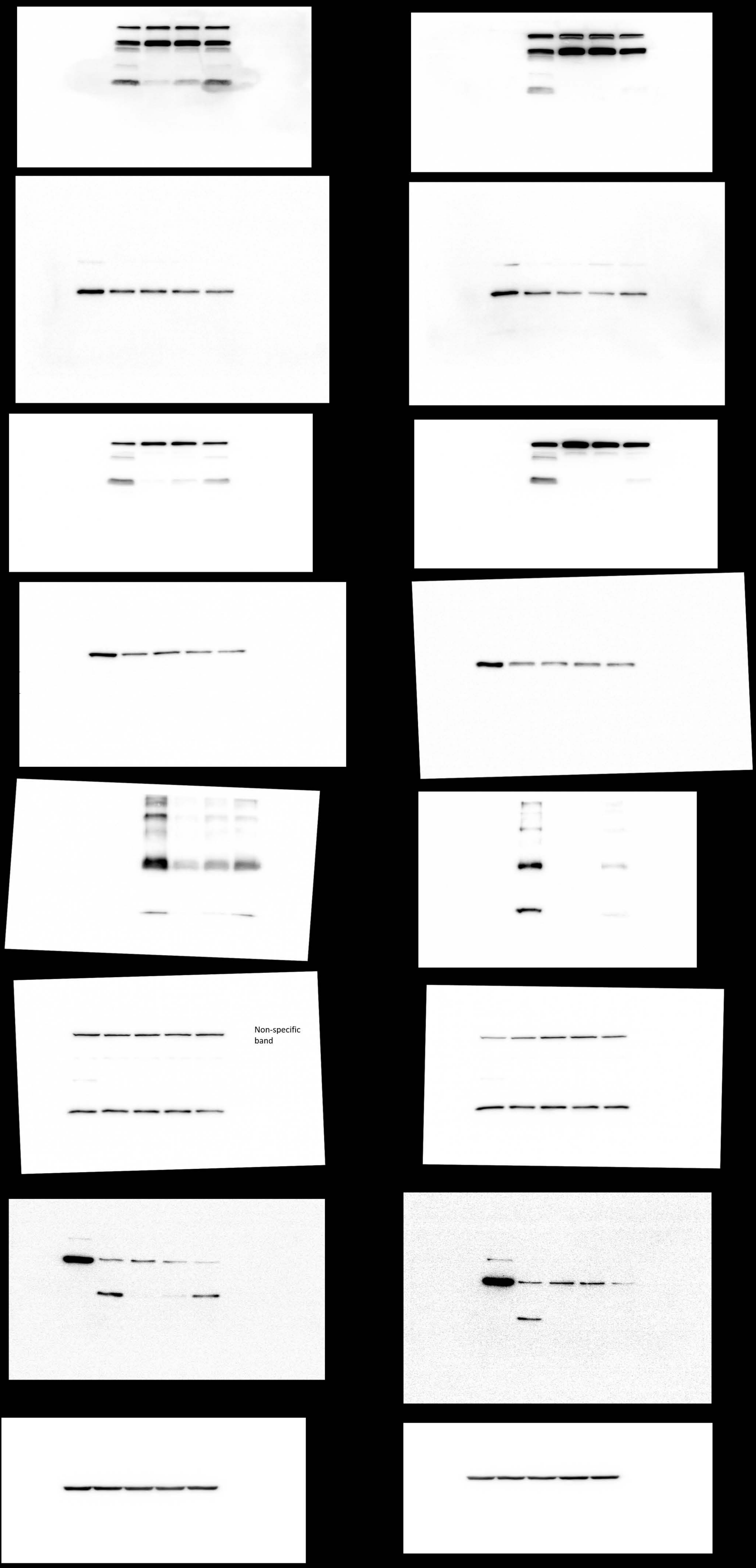

Supplement: Supplementary file 1 — Supplementary data [file 41598_2018_31717_MOESM1_ESM.pdf]
